# Cognitive–Motor Dual-Task Training (CMDT) Approaches for Performance, Recovery, Injury Prevention, Rehabilitation, and Return to Sport in Soccer: A Narrative Review with Practical Recommendations for Soccer Clubs

**DOI:** 10.3390/jfmk11020196

**Published:** 2026-05-15

**Authors:** Asaf Shalom, Roni Gottlieb, Julio Calleja-Gonzalez

**Affiliations:** 1 Department of Physical Education, The Research Center for Sports and Physical Activity, Tel Hai University of Kiryat Shmona in the Galilee, Kiryat Shmona 1220800, Israel; 2 Wingate Institute, The Academic College Levinsky-Wingate, Wingate Campus, Netanya 4290200, Israel; ronigot23@gmail.com; 3 Department of Physical Education and Sports, Faculty of Education and Sport, University of the Basque Country (UPV/EHU), 01007 Vitoria-Gasteiz, Spain

**Keywords:** soccer, cognitive–motor dual-task training, injury prevention, rehabilitation, recovery, sport-specific performance

## Abstract

This narrative review explores the potential role of cognitive–motor dual-task training (CMDT) approaches within training methods used in sports clubs, with particular emphasis on soccer clubs, to support performance enhancement, recovery, and injury prevention; improve agility, decision making, and functional readiness; and enhance training quality and specificity. The review discusses how CMDT may be integrated as part of the broader and more comprehensive planning of the club’s full training program, including during the preseason period, as part of preparation for training and competition, within recovery sessions, during periods of high load, and throughout the rehabilitation process and the transition back to team training and contact exposure, while also potentially contributing to variety, mental stimulation, enjoyment, and player engagement. The review also emphasizes the importance of implementing CMDT within a coordinated professional framework, through collaboration and synchronization within the professional and medical staff of the club, and in broad alignment with club goals, player characteristics, and sport-specific demands. The key insight is that CMDT has the potential to serve as a practical, complementary approach that helps bridge the gap between controlled training and rehabilitation settings and the dynamic demands of soccer participation. Based on this review, practical recommendations and future research directions are presented, while emphasizing that CMDT should be applied with caution, through gradual and context-specific progression, and in line with established training, recovery, and rehabilitation principles.

## 1. Introduction

Soccer is a complex sport that requires the continuous integration of physical, technical, and cognitive abilities [1,2,3]. Unlike closed tasks performed under relatively predictable conditions, soccer is performed in an open, dynamic, and rapidly changing environment, in which players are required to perceive relevant information, process it quickly, make accurate decisions, and respond according to the demands of the game [2,4]. Therefore, high-quality soccer performance does not rely only on physical fitness or technical skill, but also on the ability to integrate movement, perception, attention, and decision making under conditions of pressure and uncertainty [2,5].

Against this background, interest has been growing in the integration of cognitive–motor training and dual-task training in soccer [5]. These approaches combine a movement demand with a cognitive demand, either simultaneously or as part of a structured training sequence, with the aim of enhancing players’ ability to function effectively under conditions that are more similar to the real demands of the game [5,6,7]. The existing literature suggests that combining physical tasks with cognitive tasks may contribute both to motor performance and to cognitive abilities that are relevant for athletes, especially in aspects such as information processing speed, attention, decision making, and responses to external stimuli [6,8,9,10]. In the context of soccer, these abilities may be of particular importance in competitive situations, in which fast and accurate decisions may directly affect performance quality [5,10,11].

Beyond the possible contribution to performance, there is also growing interest in the possible role of these approaches in the context of injury prevention, rehabilitation, and return to sport [12,13]. Since many soccer injuries occur under dynamic conditions, under perceptual load, time pressure, and in response to a changing environment, assessment, training, and rehabilitation conducted only under controlled conditions may not fully reflect the actual demands of the game [14,15,16,17]. Accordingly, the integration of cognitive and movement tasks has the potential to provide a complementary means of improving players’ functional readiness, both as part of the club’s regular training program, during the preseason period, within recovery sessions and complementary training during periods of high load, and during advanced stages of rehabilitation and the transition back to team training and contact exposure [2,8,18,19,20,21]. In this context, the use of technology, together with human movement demands in changing game situations, may expand training possibilities and contribute to mental arousal, variety, creativity, and greater player engagement in the process [6,10].

### 1.1. Injuries in Soccer and the Complexity of Prevention in the Real World

Injuries in ball games, and in soccer in particular, represent a major professional and economic challenge for professional clubs because of their effect on player availability, performance quality, training continuity, and team achievements [22]. Accordingly, professional soccer clubs invest substantial resources in a broad and skilled support staff that includes professionals from coaching, sports science, medicine, and rehabilitation, based on the understanding that this investment not only improves the quality of the professional system, but may also reduce direct and indirect costs associated with injuries, time loss, and prolonged rehabilitation processes [22,23,24].

However, injury prevention in the real world is a complex process that does not rely only on physical components or on the implementation of specific exercises [22,25]. In ball games, and especially in soccer, the demand for immediate achievement, the competitive load, the density of matches and training sessions, and the need to maintain player availability throughout the season often create tension between different professional considerations [22,25,26]. At times, there is a conflict between the head coach’s desire to maximize short-term performance, the position of the medical and rehabilitation staff who seek to act with caution, the considerations of club management, and the athlete’s own desire to return quickly to training and competition [26]. Against this background, coordination among staff members and cooperation around an adapted, gradual, and realistic strategy become a meaningful component in every process of prevention, rehabilitation, and return to sport [23,25,26].

Accordingly, training strategies aimed at injury prevention also continue to develop and expand at the team, individual, and rehabilitation levels [27,28,29,30]. Within this trend, there is growing interest in the integration of cognitive training and cognitive–motor dual-task approaches, CMDT, in different variations, as part of training programs, injury prevention frameworks, and rehabilitation processes [9,13,28,30]. These approaches aim to bring preparation for training and competition, warm up and recovery strategies, the training process itself, and rehabilitation processes closer to the complex demands of actual soccer play [5,6,8].

### 1.2. Soccer as a Dynamic Environment of Perception, Decision Making, and Response to Stimuli

In ball games, and in soccer in particular, players do not act under laboratory conditions or within a predefined movement pattern, but are required to continuously scan the space, identify relevant visual information, interpret the movement of the ball, opponents, and teammates, and respond quickly to changing game situations [5,11]. Therefore, the ability to succeed in soccer does not rely only on physical and technical components, but also, to a large extent, on the ability to perceive, understand, decide, and perform the appropriate movement at the right moment [2,10].

In this context, agility takes on a broader meaning than the one sometimes measured under closed conditions [1,31,32]. Agility in soccer is not only rapid change in direction, but an open skill that combines field scanning, identification of environmental cues, immediate decision making, and sudden body adjustment to the demands of the situation [1,10]. In other words, it is not enough for the player to be fast or strong, but above all, the player must be a thinking player who is able to connect movement with real time information processing [32,33]. This ability is especially important in competitive situations, in which a small difference in reaction speed, decision accuracy, or the quality of movement adjustment may affect the success of the action and even the outcome of the game [10,32,34,35].

The previous literature on ball games also suggests that there is not necessarily a direct relationship between fast or high-quality performance under closed conditions and the ability to demonstrate similar performance under open and dynamic conditions [32,36,37]. A player who may appear very fast in a linear test or in a pre-planned drill will not always demonstrate the same quality when required to respond to an unexpected stimulus, change a decision while moving, or adjust the action to what is happening around [37,38]. This point is especially important because it emphasizes that player assessment, the development of young players, the planning of training methods at higher levels, and even decision making in processes of recovery, rehabilitation, and injury prevention should take into account the possible gap between performance under controlled conditions and performance in a real game environment [12,13,22,27,28,29,37,39,40].

### 1.3. The Gap Between Controlled Training and Rehabilitation and the Actual Demands of the Game

Despite the considerable progress in training, injury prevention, and rehabilitation, a substantial part of preparation processes, including warm up and preparation for training, training itself, and return to activity, is still carried out under relatively controlled conditions, characterized by planned practice, predictable movement demands, and a low level of uncertainty [27,41,42]. These conditions have clear importance at certain stages, especially when there is a need to establish control, accuracy, confidence, and gradual loading [27,41]. However, the recent literature increasingly indicates that an exclusive focus on muscular, mechanical, or movement components does not always reflect the full range of demands with which the player must cope when returning to the field [21,30]. In particular, there is growing understanding that the integration of cognitive components as part of the rehabilitation process may help reduce functional gaps that remain even after physical improvement, thereby supporting a safer and more relevant transition from rehabilitation to sport participation [13,21,30]. Recent studies have even proposed integrating cognitive training throughout the entire rehabilitation process, and not only in its final stages, with the aim of improving long term outcomes and reducing the risk of reinjury [43,44]. This point supports the need to consider cognitive–motor demands earlier and more progressively within the rehabilitation process.

Against this background, there is a growing need not only to restore the player to physical fitness, but also to maintain, challenge, and develop advanced cognitive components as part of the return to full sport function [21,45,46]. This rationale is especially relevant in advanced stages of rehabilitation, but also within agility training, complementary training, and dedicated injury prevention sessions, in which demands related to perception, attention, response to stimulus, and decision making during movement can be gradually integrated [6,10,45]. In this way, it may be possible to gradually reduce the gap between controlled function under rehabilitation conditions and the real demands of soccer, and to build a more complete rehabilitation process in preparation for return to team training, contact situations, and competitive performance [21,22,43,44,45].

**Figure 1:**
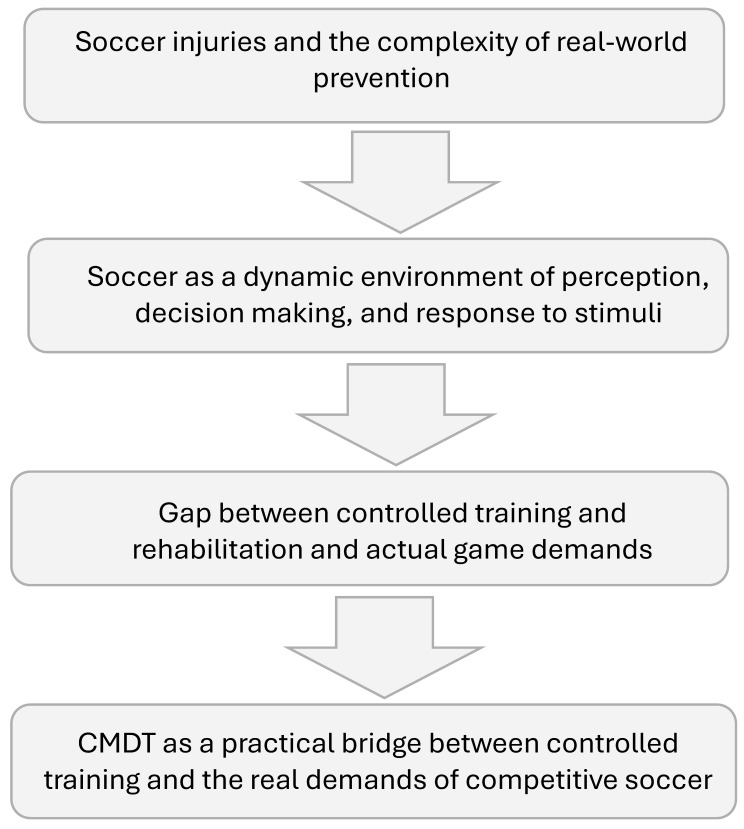
Theoretical rationale for CMDT in soccer.

As summarized in Figure 1, the rationale presented in the introduction moves from the complexity of injury prevention in real-world soccer settings, through the dynamic demands of the game and the gap between controlled training and actual match conditions, to CMDT as a practical bridge between controlled settings and competitive soccer demands. Therefore, the present narrative review aims to examine the potential role of CMDT in soccer across performance, recovery, injury prevention, rehabilitation, and return to sport contexts. Given the emerging nature of the direct evidence and the need to integrate findings from soccer and related domains, a narrative review approach was adopted to synthesize the available literature, distinguish between direct evidence and theoretical extrapolation, and provide practical recommendations for soccer clubs.

## 2. Methods

### 2.1. Search Strategy

This narrative review provides a structured and conceptually grounded synthesis of the literature related to cognitive–motor dual-task training, CMDT, in soccer. The methodological approach was designed to identify recent and relevant studies addressing CMDT, soccer performance, motor control, injury prevention, rehabilitation, return to sport, recovery, and practical implementation within the training framework of soccer clubs.

A targeted literature search was conducted across four electronic databases, PubMed, Scopus, Web of Science, and Google Scholar. The search focused on recent and relevant studies in soccer and related open skill sports, with additional foundational literature included where necessary to support the conceptual development of the topic. Search terms were developed based on professional judgment and existing literature and included combinations of the following keywords: “cognitive motor dual task”, “dual task training”, “motor cognitive training”, “soccer”, “football”, “agility”, “decision making”, “motor control”, “injury prevention”, “rehabilitation”, “return to sport”, “recovery”, “warm up”, and “sport performance”. These terms were adapted across databases to ensure broad coverage. In addition, reference lists of relevant articles were screened to identify further studies of interest.

### 2.2. Study Selection and Conceptual Scope

Studies were considered for inclusion if they addressed cognitive, motor, psychomotor, neurocognitive, or integrative training approaches relevant to soccer performance, injury prevention, rehabilitation, return to sport, or recovery. Studies conducted in soccer were prioritized when available. Given that direct evidence on CMDT in soccer remains limited in some areas, the review also included studies from related domains, such as dual-task training, motor cognitive training, neurocognitive training, agility training, chronic ankle instability, ACL rehabilitation, warm up interventions, recovery strategies, and return to sport research, where they provided meaningful insight into the interaction between cognitive and physical demands.

These related domains were included because the current literature does not yet provide a complete evidence base for CMDT across all soccer contexts. Therefore, evidence from adjacent areas was used to support a broader conceptual understanding of how cognitive–motor demands may relate to performance, motor control, injury prevention, rehabilitation, recovery, and return to sport. Throughout the review, an effort was made to distinguish between direct evidence from soccer or CMDT studies and theoretical extrapolation from related fields.

The selection of sources was guided primarily by their relevance to the main themes of the review, rather than by a strict methodological hierarchy. Empirical studies, randomized and controlled interventions, review papers, and theoretical or applied frameworks were considered when they contributed to the aims of the review. Review papers were included only when they helped clarify existing concepts, summarize relevant evidence, or identify gaps that justified the need for the present narrative synthesis.

### 2.3. Data Synthesis and Thematic Organization

The synthesis of the literature followed a thematic approach, in which studies were grouped according to key topic areas relevant to CMDT in soccer. These areas included performance and sport-specific training, motor control and injury prevention, rehabilitation and return to sport, recovery and readiness, and practical implementation within the training framework of a soccer club.

These topic areas were selected because they reflect the main contexts in which CMDT may have practical relevance in soccer. Performance and sport-specific training were included because CMDT may help examine and develop actions performed under perceptual and decision making demands. Motor control and injury prevention were included because injury risk in soccer often emerges during dynamic actions that require rapid perception, decision making, and movement adjustment. Rehabilitation and return to sport were included because the transition from controlled rehabilitation to team training and competitive play requires not only physical recovery, but also the restoration of cognitive–motor function under soccer-specific conditions. Recovery and readiness were included because emerging evidence suggests that motor cognitive activity may influence subsequent reactive performance and may be relevant within broader load management strategies.

This approach aimed not only to summarize existing findings, but also to identify gaps in the literature and support the development of a broader conceptual framework for the application of CMDT in soccer. In particular, the review sought to clarify where the evidence is direct, where it is emerging, and where recommendations are currently based mainly on theoretical or mechanistic reasoning. This distinction was considered important for developing practical recommendations while maintaining appropriate caution regarding the current state of the evidence.

## 3. CMDT in Soccer, Implications for Performance, Motor Control, and Injury Prevention

In soccer, players are required to execute movement actions while continuously processing information and responding to changing stimuli [5,7]. Therefore, CMDT provides a relevant framework for examining performance under more game-like conditions [2,7,10]. This section therefore focuses on the main CMDT approaches used in soccer and related ball games, including agility tasks combined with visual stimuli, dribbling or technical actions under cognitive load, tracking tasks, calculation tasks, and reactive decision making tasks. Current evidence from soccer and related ball games indicates that motor–cognitive training interventions can improve agility, technical performance, and sport-specific outcomes, particularly when players are assessed under dual-task or reactive conditions [5,6,18,46,47].

Accordingly, the rationale for integrating CMDT in soccer is based on the understanding that agility, motor control, and high-quality performance are not the result of physical components alone, but also of the ability to function under cognitive load, uncertainty, and response to external stimuli [1,10,11,48,49]. In this context, CMDT may help sharpen responsiveness, improve decision making quality, and strengthen the relevance of training to the sport-specific demands of soccer [2,5,9,50]. From an injury prevention perspective, the rationale for CMDT is mainly mechanistic and theoretical at this stage. Many risk situations in soccer do not occur under closed or predictable conditions, but rather during actions that require rapid perception, immediate movement adjustment, and decision making under pressure [21,45]. Therefore, an approach that combines movement with cognitive demand may better reflect the conditions under which players are required to perform [6,43,44,45]. However, direct evidence showing that CMDT reduces injury rates in soccer is still limited. In the present review, injury prevention is therefore discussed mainly on the basis of motor control, postural control, balance, agility, and neurocognitive mechanisms that may be relevant to injury risk.

The recent literature also suggests that CMDT may be effective when integrated into training programs, including during relatively short intervention periods, and as part of warm up and preparation routines [5,7,8,9,11]. These findings support the view that cognitive–motor training is not only a theoretical addition to soccer training, but also an applied approach with potential to improve functional readiness and performance quality when it is implemented with appropriate task design and progression [10,45]. Beyond this, CMDT may also be relevant in the context of recovery, rehabilitation, and return to sport after injury, since these processes do not end with the restoration of physical ability alone, but also require the gradual return of attention, perception, response to stimulus, decision making, and motor control under conditions that are closer to the reality of the game [13,21,45]. Against this background, CMDT may be viewed as a complementary approach with a clear theoretical rationale, linking performance enhancement, motor control, injury prevention, recovery, rehabilitation, and return to full sport function in ball games, and in soccer in particular [5,11,12]. At the same time, the current evidence base is not equally developed across all these areas. Therefore, the following sections distinguish between direct evidence, emerging evidence, and theoretically grounded applications.

These aspects will be further expanded in the next section, which will focus on the possible contribution of CMDT to rehabilitation and return to sport processes.

## 4. CMDT in Rehabilitation, Return to Sport, and the Transition Back to the Demands of Soccer

The current evidence base is not equally developed across all injury types. The strongest rationale for integrating CMDT appears mainly in relation to ACL injury and chronic ankle instability, where broader literature exists on motor control, neurocognitive demands, balance, and return to sport. In hamstring injuries, an emerging research direction also supports the relevance of cognitive–motor considerations, particularly in relation to high-speed running, deceleration, fatigue, and return to sport decision making. In contrast, recommendations related to meniscus and other possible injuries are more indirect, and are mainly extrapolated from the literature on motor control, performance, gradual exposure, and return to sport criteria.

### 4.1. Cognitive–Motor Changes After Injury

After injury, and especially after major ACL injuries, rehabilitation of strength, range of motion, and functional symmetry alone is not always sufficient [43,44,45]. The recent literature suggests that throughout the rehabilitation process, deficits may remain in attention, reaction speed, motor planning, neuromotor control, and reliance on visual feedback, even when physical measures improve [12,13,45,51,52]. Therefore, the rationale for integrating CMDT in rehabilitation is based on the understanding that return to sport, and in soccer in particular, should reflect not only physical readiness but also the ability to function under external stimulus, uncertainty, and time pressure. Accordingly, the literature has proposed integrating cognitive–motor challenges throughout the rehabilitation continuum in order to reduce the gap between controlled function in the clinic and the actual demands of the field [21,30,45].

### 4.2. Gradual CMDT Integration During Rehabilitation

In the earlier stages of rehabilitation, cognitive components should be integrated only when there is already an initial level of good control of the exercise, stable performance, and appropriate loading [45]. At this stage, the intention is not to create chaos too early, but rather to gradually add simple and controlled tasks, for example visual tracking, response to a color or sound, counting, basic working memory, simple ball passing, or choosing between two options while performing a controlled exercise [43,44,45]. From an applied perspective, such integration may also add value to the rehabilitation process, increase involvement and interest, and maintain cognitive–motor functional continuity, as long as it is built properly, well-controlled, and does not impair movement quality [10,13,45].

In the more advanced stages, toward return to running, changes in direction, team training, and contact situations, there is an even stronger rationale for integrating CMDT [6,45]. At this stage, it is possible to gradually progress to exercises that include response to a stimulus, visual search, decision making, reactive change in direction, landing or jumping with a cognitive task, and agility challenges in which the player is required to think and move at the same time [12,43,44,45,51]. In the context of ACL, several recent articles even recommend expanding return to sport testing beyond physical measures alone, and integrating cognitive–motor challenges in order to identify neural compensations that may remain hidden in standard testing [13,21,30,45,53].

### 4.3. Injury-Related Evidence and Practical Considerations

From an injury-specific perspective, the strongest direct basis at present is found around ACL, where both the early integration of visual and cognitive stimuli in simple and controlled exercises, and the more advanced integration of reactive, visual, and movement tasks before full return to sport have been described [43,44,45]. In the ankle as well, and especially in cases of chronic instability, the literature suggests that dual-task training may improve static and dynamic balance, and therefore provides a good example of CMDT integration in single-leg-control situations, balance tasks, landings, and responses to external perturbation [12,27,51]. For hamstring injuries, the direct evidence for CMDT remains more limited than for ACL and chronic ankle instability, but the emerging literature suggests that cognitive–motor demands may be relevant, especially during high-speed running, fatigue-related situations, decelerations, changes in direction, and return to sport decisions under time pressure [54,55,56]. In contrast, in meniscus injuries and other possible injury contexts, the direct basis for CMDT is still more limited, but the return to sport literature emphasizes the need for performance-based criteria, gradual exposure, and decision making matched to the level of complexity [29,57]. Therefore, careful integration of CMDT may also be justified in these cases, mainly in the later stages, for example during high-speed running, decelerations, changes in direction, response to a stimulus, and advanced technical exposure after control, range of motion, strength, and load tolerance have been restored [6,45].

Therefore, CMDT may be viewed as a complementary approach within modern soccer rehabilitation, not as a substitute for the basic principles of control, loading, strength, and gradual progression, but as an additional component that brings the player closer to the real demands of returning to the field [7,58]. Its possible value is especially evident when it is integrated in a stage-appropriate way, from simple and controlled tasks after the establishment of basic control, to more complex and reactive tasks in the advanced stages of rehabilitation and return to contact [45,58].

## 5. Integration of CMDT Within the Training Framework of a Soccer Club

Structured injury prevention, warm up, recovery, and rehabilitation processes are central parts of the professional training framework in soccer clubs [11,39,41,59,60,61]. Within this framework, CMDT may offer an additional way to connect physical preparation with perception, attention, response to stimulus, and decision making during movement. The purpose of this section is to outline practical principles for integrating CMDT within the soccer club environment, while recognizing that some applications are supported by direct or related evidence, whereas others should be viewed as theoretically informed directions that require further investigation.

### 5.1. CMDT as Part of the Routine Training Program in the Club

CMDT is not only a rehabilitation tool, but also a component that can be integrated into the club’s routine work [5,6,9,11]. The rationale is not to build separate and long units in every training session, but rather to integrate short, gradual, and specific parts within the team program [9,10,11,47,62]. For example, these may be included during warm up, at agility stations, in passing and decision making drills, in response to stimulus tasks, and in short parts of technical work under a perceptual demand [8,10,63]. The soccer literature supports the idea that a short and planned integration of cognitive–motor training within the training routine may improve agility measures, cognitive technical performance, and dual-task costs, and is therefore highly suitable for routine work in the club. In addition, the recent literature on the neurocognitive enrichment of warm up and injury prevention supports the idea that such integration may also be more feasible and more attractive for players [8,10,27,41,64].

### 5.2. Integration During the Preseason Period

In this period, CMDT should be integrated mainly through warm up, short parts within field training, and agility and response drills under gradual load [65,66,67]. It is important not to turn the preseason period into an additional cognitive load without control, but rather to use CMDT to improve functional readiness for training, raise performance quality, and connect physical loads with demands related to scanning, response, and decision making [6,8,66,68].

It is important to note that the direct literature on CMDT specifically within the preseason period in soccer is still limited. Therefore, the present rationale draws on evidence from short-duration CMDT studies, the warm up literature, and the broader literature highlighting the importance of a gradual preseason progression for reducing injury risk.

### 5.3. CMDT Within Recovery Sessions and High Load Periods

During periods of high load, it is not always appropriate to add high-load physical sessions, but CMDT can still be integrated under low to moderate movement load in order to maintain movement sharpness, interest, variety, and process quality [69,70,71,72]. For example, this may include short units or sessions involving visual response, passing and decision making under low-load, balance and response drills, or simple technical stations with an attentional demand [7,70,71]. The possible advantage is that the player receives a functional and mental stimulus even when there is a need to exercise extra caution during periods of high training load [6].

This is especially important because the literature shows that congested periods, international travel, and match loads are associated with increased injury susceptibility, and that residual fatigue may still be present even after 72 h, especially in muscles such as the hamstrings [20,73,74,75,76,77,78]. Therefore, during such periods, CMDT has the potential to serve as a complementary approach for maintaining readiness, as long as relatively simple and short tasks are selected and carefully synchronized with the professional content of the training program and with the professional staff [6,23,25,26].

### 5.4. Agility, Decision Making, and Sport-Specific Training Quality

CMDT may improve not only physical performance, but also the quality of training, because it brings practice closer to the actual demands of soccer [10,79]. The soccer literature already shows that specific CMDT interventions can improve agility, cognitive flexibility, technical performance under visual interference, and decision making under pressure [5,11]. Beyond this, previous studies have shown that cognitive–motor agility tests are more strongly related to cognitive measures and indirect game-related measures, compared with more closed tests [10,31,32,79,80,81,82].

Therefore, CMDT does not only add difficulty, but may also enhance the quality of practice so that it becomes more creative, more applied, and more specific to the demands of the game [6,7,10].

### 5.5. Considerations for High-Quality CMDT Implementation in a Soccer Club

Not every exercise that includes light, color, sound, or another external stimulus necessarily represents a high-quality implementation of CMDT [6]. Similarly, not every combination of cognitive and movement demand is suitable for every age group, every player level, every training session, or every phase of the season [6,67,83,84]. Therefore, the quality of CMDT implementation depends on precise adjustment to the purpose of the session, the load planned for that day, the level of the players, the phase of the season, the rehabilitation status if relevant, and additional team characteristics, including player age, level of professional maturity, and the team’s mental state [21,69,70,71].

Beyond this, the injury prevention literature consistently emphasizes that the success of applied programs depends not only on the content of the practice itself, but also on the quality of implementation, the level of player adherence, the simplicity and clarity of the instructions, and the degree of cooperation among staff members [22,24,25]. Within a soccer club, CMDT implementation is likely to be more effective when the head coach, strength and conditioning coach, physiotherapist, sports scientist, and medical staff work from a shared load management approach, clear professional goals, and a unified professional language [22,26]. This approach may support the gradual, context-appropriate, and feasible integration of CMDT within the training, prevention, and rehabilitation program [22,23,25].

**Figure 2:**
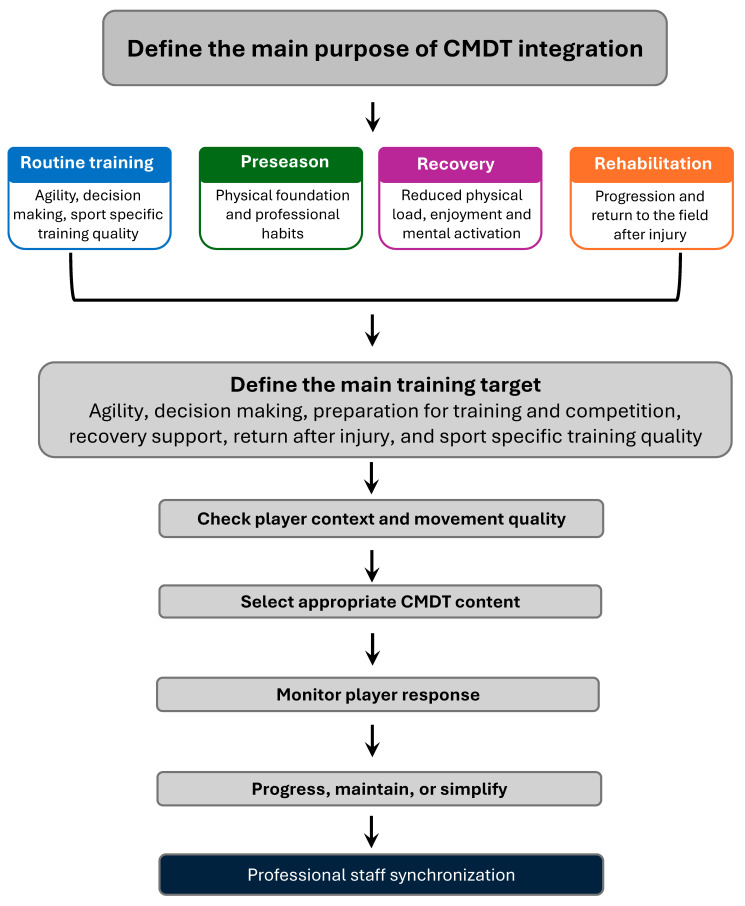
Flowchart of possible considerations for CMDT integration in a soccer club.

Given the complexity of implementing CMDT in a soccer club, Figure 2 presents a proposed flowchart intended to facilitate a deeper understanding of possible considerations for integrating CMDT according to the purpose of the session, the player context, and the player’s functional stage. These considerations are also presented in a more applied way in the concluding section of this narrative review.

## 6. Discussion

The main aim of this narrative review was to examine the possible value of CMDT for performance, recovery, injury prevention, rehabilitation, and return to sport in soccer, while emphasizing its relevance within the framework of training programs in a soccer club. The literature discussed suggests that CMDT should not be viewed as a substitute for the accepted principles of training, injury prevention, and rehabilitation, but rather as a complementary component that may strengthen the specificity, practical value, and connection to the real demands of ball games, and of soccer in particular. In this sense, the possible contribution of CMDT lies not only in adding cognitive load, but also in its ability to bring the training, preparation, recovery, and rehabilitation environment closer to the open, dynamic, and context-dependent demands that characterize the game in practice [5,18,85,86]. At the same time, because the direct evidence is not equally developed across all these areas, the discussion adopts a cautious interpretation and frames some applications as theoretically informed directions that require further investigation.

The literature also supports consideration of the perception of cognitive effort [87], which may also be particularly relevant during periods of tapering and reduced training load [60,70,88,89]. Recent evidence further suggests the view that active motor cognitive recovery supports subsequent reactive agility performance [20]. The existing literature also supports the view that cognitively challenging training has greater potential value when cognitive demands are integrated within sport-related movement contexts, rather than addressed through isolated computer-based tasks that show limited transfer to real sport performance [90].

In the context of performance enhancement, the recent literature provides stronger evidence that integrating CMDT within training programs contributes to improvements in agility, cognitive–motor function, and the quality of sport-specific performance. The importance of this finding is especially evident in open skill sports, in which closed and isolated physical abilities alone are not sufficient to explain the quality of function under changing conditions [2,7,9,11,50,62,91]. Therefore, one of the main messages emerging from the literature is that it is not appropriate to assume that an individual who demonstrates higher performance in closed and pre planned situations will necessarily maintain the same advantage in open situations that require response, scanning, information processing, and decision making [37]. In addition, previous studies have shown that CMDT can also be effective when integrated with other training methods, such as multi component training (MCT), which opens the door to further practical combinations within the broader training program [7,9].

From an applied perspective, the literature also suggests that CMDT may have practical potential for integration within warm up routines designed to support performance preparation, as well as within rehabilitation and return to sport processes after injury [6,7,8,63]. Recent studies in ball games suggest that the inclusion of cognitive challenges within warm up may immediately improve readiness for performance, thereby contributing to better functional preparation for training and competition [8]. At the same time, the recent ACL rehabilitation literature emphasizes that return to the field should not rely only on physical measures, because after injury, deficits may still remain in information processing, motor planning, neuromotor control, and dependence on visual feedback [13,45,53]. This supports a clear rationale for integrating cognitive components already in the early stages of rehabilitation, but only after the establishment of basic control and under controlled conditions, and then gradually progressing toward more complex tasks that include response to stimulus, attention, decision making, and movement, in preparation for return to team training, chaotic situations, and contact [43,44,45].

Alongside the potential of CMDT, it should be emphasized that its integration within injury prevention sessions, recovery sessions, and final return to sport protocols before return to contact has still not been sufficiently studied. At the same time, the existing literature already provides an adequate basis for viewing CMDT as a promising applied and research direction, especially when it is integrated as an integral part of team-based work rather than as an isolated tool [5,7,10,18].

In this regard, it appears that the most meaningful progress in the future will not come only from examining individual exercises, but from the development of systematic, gradual, and context-appropriate methodologies, from the preseason period, through injury prevention and recovery units, to inclusion in rehabilitation and return to sport protocols. Such a process also requires full coordination among the head coach, strength and conditioning coach, physiotherapist, sports scientist, and medical staff, with adaptation to the team mentality, the age of the players, their level, and their characteristics. If CMDT is indeed expected to become a regular strategy in soccer clubs rather than a developing mechanism in the field, there is a need to begin its implementation already at younger ages and to continue investigating the ideal dose, timing, and way to integrate it within the broader training program [21,22,24,25,26,68,70,71].

## 7. Practical Recommendations and Future Research Directions

### 7.1. Practical Recommendations

The current review suggests that the integration of CMDT approaches may have real applied value when it is developed in a gradual, systematic, and context-appropriate way within the professional setting of a soccer club. Accordingly, CMDT should be viewed as part of a broad and continuous process, from the preseason period as a foundation for developing a shared professional language, through its integration within warm up routines as preparation for training and competition, within recovery sessions, in dedicated injury prevention sessions, and also in advanced stages of rehabilitation and return to contact and full competitive function. High-quality implementation of this approach requires continuous adjustment to the purpose of the session, the daily load, the phase of the season, the level of the players, and their mental and functional status.

In this context, it is highly important to integrate CMDT within a periodization framework and load planning across the season, in accordance with the demands of the game, and especially in elite competitive soccer, where periods of high load, match congestion, and physical and mental fatigue are common. In such situations, CMDT has the potential to be applied in soccer settings as a complementary component that allows for, on the one hand, a controlled reduction in physical load, and on the other hand, the maintenance, refinement, and continued development of cognitive–motor abilities, alongside a contribution to mental refreshment, variety, and preservation of training process quality in line with the broader plan.

Beyond this, successful implementation of CMDT in the field does not rely only on professional knowledge, but also on applied skill, creativity, and the ability to design practice that is varied, precise, and relevant to the demands of soccer. In this context, proficiency in technology may expand the possibilities for implementation, but it should not be viewed as a goal in itself, but rather as an additional means within a broader framework of work, based first and foremost on human movement, performance quality, and a deep understanding of the aims of training and rehabilitation.

Alongside this, full coordination among the professional staff is of great importance. A lead professional, such as a sports scientist or another professional with an integrative perspective, may help shape the professional direction, support coordination with the head coach and the entire professional and medical staff, and facilitate the consistent implementation of CMDT as part of the club’s working structure.

### 7.2. Future Research Directions

Given the potential of CMDT, further studies are needed to examine its effectiveness as part of systematic implementation across a full season, rather than only within short interventions or isolated experiments. There is also a need for more research on its possible contribution during the preseason period, within warm up protocols, as part of recovery strategies, within dedicated injury prevention sessions, and in the final stages of return to sport and contact. In addition, it is important to examine how CMDT implementation can be adapted according to age, player level, playing position, training background, mental characteristics, and different types of injuries, including the distinction between short-term injuries and more prolonged and complex injuries.

Comprehensive future research is needed to examine the possible effectiveness of CMDT within periodization and load management processes across the season, especially in elite competitive soccer. In particular, it is important to examine whether the planned integration of CMDT during periods of high load may help maintain cognitive–motor abilities, reduce accompanying physical load, and support better adaptation to the changing demands of the training process. The ideas presented by our research group in this article also open the door for future research on the integration of CMDT within tactical periodization across the weekly training cycle and across different phases of the season, from preseason periods to competitive phases under demanding match schedules. Future studies should examine whether CMDT dosage should vary according to the structure of the training week, timing in relation to competition, and the need to maintain neuromuscular freshness. In this context, it remains important to examine whether CMDT should be integrated two days before the match, when the priority is often to maintain neuromuscular freshness, or whether such timing may impose excessive cognitive demands.

Another research direction that may be of interest relates to cognitive and movement stimuli. There is a need to examine more systematically the use of technology compared with human movement, as well as the possible contribution of combining the two. It is also appropriate to examine the effects of background noise, environmental load, pressure situations, and uncertainty on performance quality and on rehabilitation and return to sport processes. Finally, it is important to expand the research to comparisons across different sports, in order to better understand which CMDT principles require more specific adaptation to the unique demands of ball games in general, and soccer in particular.

## 8. Conclusions

Current evidence indicates that CMDT can enhance cognitive–motor performance and sport-specific outcomes, particularly under dual-task and reactive conditions, when implemented within structured training programs. CMDT may therefore be considered a complementary component with potential for performance enhancement, recovery, injury prevention, rehabilitation, and return to sport in soccer, especially when it is implemented as part of a specific, adapted, and careful training program. The applied potential of this approach may be more evident when it is developed as part of a broader systematic strategy, from the preseason period as a foundation for practice, through warm up routines, recovery strategies, injury prevention training, and rehabilitation processes, and up to return to full sport function.

However, high quality implementation of CMDT likely requires full coordination among the professional staff, adjustment to the team context and player characteristics, and thoughtful integration based on established physiological, biomechanical, and rehabilitation principles. Therefore, CMDT should be considered not as a substitute for accepted principles, but rather as a complementary component with practical potential, one that may require creativity and applied skill on the one hand, and deep understanding, professional precision, and gradual planning on the other, within soccer clubs and related team sport environments.

## Data Availability

No new data were created or analyzed in this study. Data sharing is not applicable to this article.

## Abbreviations

The following abbreviations are used in this manuscript:

CMDT	Cognitive–motor dual-task training
MCT	Multi component training
